# Books and Magazines

**Published:** 1898-05

**Authors:** 


### Books and Magazines.

No one who is interested in the best contemporary French literature can afford to miss the series of sketches and stories by Paul Bourget, which will begin in *The Living Age* for April 2. These sketches have been but recently published in France, and this is their first appearance in English dress. They are translated for *The Living Age* by William Marchant. They are extremely clever and characteristic.

---

Mrs. Cleveland's New Portraits.—Mrs. Cleveland recently had a new set of photographs taken, the first time she has been photographed since leaving the White House, and has given them to Mr. Bok, with permission to publish them in *The Ladies' Home Journal*, where they will be publicly seen for the first time. The set also includes the first authoritative photographs published of the new Princeton home of the Clevelands.

IN HISTORY-MAKING times like these a truthful record of passing events becomes an imperative need. The daily newspaper is ephemeral and not easily preserved for reference. The *American Monthly Review of Reviews* has all the value of the newspaper besides distinctive merits of its own. As an epitome of current history it is complete, compact, terse, impartial, absolutely reliable, and judiciously edited. As a piece of journalistic history-writing what could be more brilliant or facinating than the May number of this publication, with its story of the Spanish-American war-crisis? Merely a souvenir of the past eventful month the *Review* has a certain unique fitness.

